# Suitability of Short- and Long-Term Storage of Volatile Organic Compounds Samples in Syringe-Based Containers: A Comparison Study

**DOI:** 10.3390/metabo13080903

**Published:** 2023-08-02

**Authors:** Paulo Henrique Costa Santos, Pedro Catalão Moura, Valentina Vassilenko

**Affiliations:** 1 Laboratory for Instrumentation, Biomedical Engineering and Radiation Physics (LIBPhys-UNL), Department of Physics, NOVA School of Science and Technology, NOVA University of Lisbon, Campus FCT-NOVA, 2829-516 Caparica, Portugal; pr.moura@campus.fct.unl.pt; 2 NMT, S. A., Edifício Madan Parque, Rua dos Inventores, 2825-182 Caparica, Portugal

**Keywords:** air storage containers, syringe, volatile organic compounds, gas chromatography, ion mobility spectrometry, breath sampling

## Abstract

The employment of advanced analytical techniques and instrumentation enables the tracing of volatile organic compounds (VOCs) in vestigial concentrations (ppbv-pptv range) for several emerging applications, such as the research of disease biomarkers in exhaled air, the detection of metabolites in several biological processes, and the detection of pollutants for air quality control. In this scope, the storage of gaseous samples is crucial for preserving the integrity and stability of the collected set of analytes. This study aims to assess the suitability of three commercially available syringes as air containers (AC) that are commonly used for the collection, storage, isolation, and transportation of samples: glass syringes with glass plungers (AC1), and two plastic syringes, one with plastic plungers (AC2), and one with rubbered plungers (AC3). For this purpose, 99 air samples with different times of storage (from 10 min to 24 h) were analyzed using a Gas Chromatography—Ion Mobility Spectrometry device and the degradation of the samples was properly assessed by comparing the changes in the VOCs’ emission profiles. The quality of the method was assured by via the measurement of the blank’s spectra before each experimental run, as well as by the consecutive measurement of the three replicates for each sample. A statistical analysis of the changes in the VOCs’ emission patterns was performed using principal component analysis (PCA). The results, with a total explained variance of 93.61%, indicate that AC3 is the most suitable option for the long-term storage of air samples. Thus, AC3 containers demonstrated a higher capacity to preserve the stability and integrity of the analytes compared to AC1 and AC2. The findings of the short-term effects analysis, up to 1 h, confirm the suitability of all analyzed syringe-based containers for sample-transferring purposes in onsite analysis.

## 1. Introduction

The storage and analysis of volatile samples has emerged as an expanding field of high interest, providing valuable insights into metabolite composition, characteristics, and sources. Understanding the nature and origin of the compounds present in these samples, which generally include over 300 different volatile organic compounds (VOCs) in a large range of concentrations (from ppm_v_-ppt_v_), is crucial for identifying their sources and potential impact in numerous applications, including environmental monitoring (or air quality monitoring) and disease detection using exhaled air. VOCs are characterized by their low boiling points, which allow them to easily evaporate into the gaseous phase, and originate from both endogenous and exogenous sources. Endogenous VOCs are produced in living organisms and can be indicative of metabolic processes or diseases, whereas exogenous VOCs are derived from external sources [1] such as industrial emissions, vehicle exhausts, or natural processes, e.g., food [2] and plant emissions.

Since the analysis of volatile samples highly depends on robust storage and analysis methods, there is a need to employ advanced analytical techniques that enable the application of gas analysis for healthcare (i.e., screening multiple health conditions/diseases using exhaled air [3,4,5,6] and air quality control applications [7,8,9,10,11]). Environmental monitoring involves the assessment of air quality in various settings, such as industrial sites, urban areas, and indoor environments, to ensure compliance with regulations and to evaluate potential health risks [12]. Additionally, the detection of diseases using exhaled air, known as breath analysis, has gained significant attention in recent years. Studies have shown that certain diseases, including lung cancer and respiratory infections, can be detected by analyzing specific VOC profiles in breath samples [6,13,14]. In this scope, gas chromatography–ion mobility spectrometry (GC–IMS) has emerged as a powerful analytical technique that offers advantages due to its ability to perform fast and cheap analysis, its high sensitivity, and its ability to detect a wide range of compounds, making it a valuable tool for such applications [15].

While volatile sample analysis provides valuable information, it is essential to consider the limitations associated with the transfer and storage of these samples. One major limitation is the potential loss or reduction in VOCs’ concentration during the transfer process. This can occur due to the adsorption of compounds into the internal walls of transfer lines or their diffusion through the walls of the collection vessel. Hence, a suitable approach to air sample storage would enable the preservation of the integrity and stability of collected analytes, leading to an accurate analysis of pollutants and disease biomarkers [16,17].

A specific limitation occurs when gas samples pass through transfer lines, where certain compounds can be adsorbed onto the internal walls, resulting in a loss of concentration [18]. This phenomenon is particularly significant for VOCs, as they tend to have higher affinities for solid surfaces. The extent of adsorption can vary depending on factors such as the length and composition of the transfer line material and the physicochemical properties of the compounds involved. Besides the concentration loss of several VOCs due to adsorption, the contamination of gas samples also occurs due to reactivity between the compounds and the materials (storage vessels and/or transfer lines). Some VOCs may react with contact surfaces, leading to chemical reactions that may generate new compounds that alter the composition of the gas samples and introduce artefacts into the analysis [19,20].

To overcome the limitations associated with gas sample transfer and storage, various types of storage containers have been used [21,22]. These include Tedlar bags, sorbent tubes, needle trap devices, and syringes. Each container type offers distinct advantages and disadvantages related to cost, sample preservation and compatibility with analytical techniques. Syringe-based containers are the least costly sample container capable of storing air samples agnostically (i.e., non-specific to predetermined VOCs), and have internal walls with a reduced surface area compared to other containers and typical PTFE-based bags. The smaller surface area minimizes the adsorption of sample VOCs onto the walls, thus preserving the sample’s composition more effectively, as well as avoiding sample contamination due to degassing from the wall surface [23,24]. This feature is very important for short-term sample storage and transferring a sample into the analyzer.

There are multiple syringe-based containers commercially available with distinct characteristics that may impact VOCs’ emission profile and, thus, sample composition. Moreover, a temporal characterization of the sample stability of syringe-based containers is still missing. Therefore, and considering the importance of gas sample analysis and the limitations associated with sample transfer and storage, this study aims to achieve two objectives: (1) to verify the maintenance of the VOC profile (i.e., sample composition) when air samples are trapped for a short period (solely for the purpose of transferring a sample to the analyzer) in three different types of syringes, and (2) to evaluate the temporal capacity (up to a maximum of 24 h) with which the VOC profiles for air samples stored in different syringes are maintained. Summarily, this study involves assessing how the composition of air samples changes over time when stored in various syringe-based containers. By evaluating the temporal stability of samples, this study aims to provide insights into the optimal storage duration and syringe type for preserving sample integrity. The authors expect that the findings presented in this study will contribute to improving the quality and reliability of gas sample analysis and facilitate advancements in various applications relying on gas composition data.

## 2. Materials and Methods

### 2.1. Air Storage Containers

**Figure 1:**
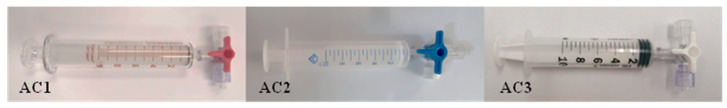
Syringe-based Air Containers (AC) used in the study: full glass (AC1, **left**), plastic with plastic plunger (AC2, **middle**) and plastic with rubbered plunger (AC3, **right**).

Three types of syringe-based containers with different compositions (glass and plastic with and without rubbered plungers) were selected and evaluated for their suitability to store air samples. Sterile 10 mL volume containers were used in every case. The glass syringes (AC1) were manufactured by Poulten & Graf^®^, the plastic syringes with plastic plungers were produced by BD^®^ (AC2), and the plastic syringes with rubbered plungers (AC3) were manufactured by PIC Solution^®^. The sterile condition of each container was ensured by the manufacturers. All the containers were acquired via officially recognized distributors. Figure 1 displays the 3 types of air containers used throughout the study.

### 2.2. Sample Collection and Storage

Environmental air samples from a controlled closed space (i.e., a laboratory facility) were considered for this study. One single operator collected and analyzed the samples to minimize experimental variability among the rounds of sampling. Air samples of 5 mL were collected into sterile containers by applying negative pressure as a result of the forced movement of the plunger and were then constrained using three-way valves. Subsequently, syringes were stored in a freezer at 8 °C and left exposed to the same conditions of temperature, pressure, humidity, and radiation until the moment of analysis.

Three air sample replicates per container type were collected and analyzed over 24 h in several instances, i.e., 0 min, 10 min, 30 min, 1 h, 2 h, 4 h, 8 h, 12 h, 16 h, 20 h, and 24 h after their collection and isolation. Therefore, a total of 99 air samples were included in this evaluation (specifically, 11 measurements were performed for each container, totaling 33 spectra for 3 container types). 

### 2.3. Sample Analysis

#### 2.3.1. IMS-Based Technologies

Ion Mobility Spectrometry (IMS) is an analytical technique that has been gaining relevance in several scientific fields, namely environmental, health, and food research applications [25,26,27]. Its main characteristics, namely its outstanding sensitivity, high selectivity, instrumental simplicity, analytical flexibility, portability, and almost-real-time monitoring capacity, have placed IMS among the most promising separation techniques for the assessment of VOCs. If coupled with a chromatographic column, the resulting device couples these features with good precision, a wide dynamic concentration range, and high Gas Chromatography (GC) selectivity, with improved capacities to differentiate VOCs considering their size, weight, and molecular shape [12,15,28].

**Figure 2:**
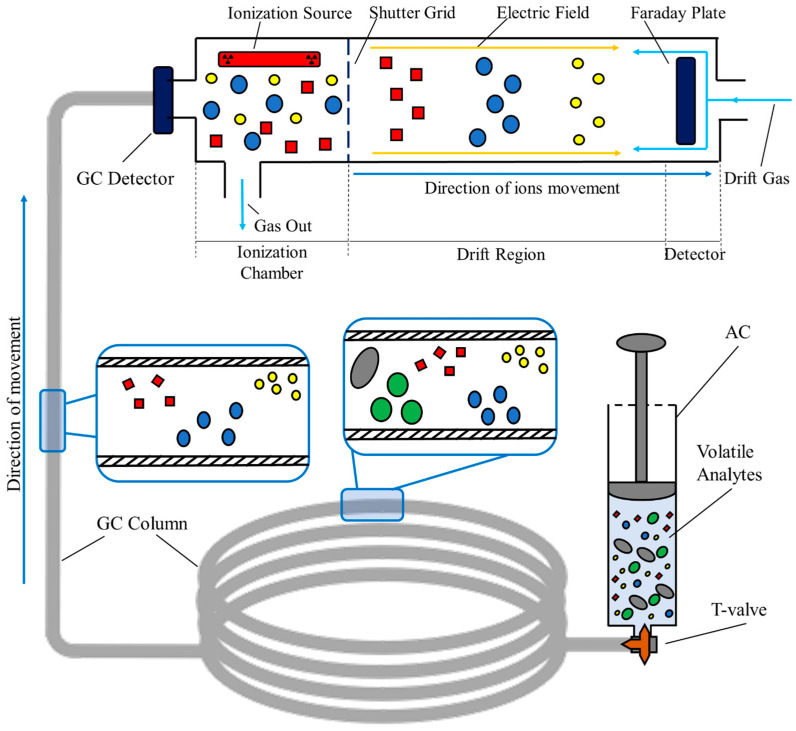
Setup for sampling and analyzing process of room air samples collected in syringe-based air containers via GC–IMS.

In summary, a GC–IMS measurement starts with the injection of the volatile sample into the spectrometer. Here, the sample undergoes a pre-separation process in the interior of the chromatographic column, where the analytes are separated based on their capacity to absorb into the inner coating of the column. The time required to completely elute from the GC is a compound-specific value and is commonly defined as the retention time, rt. This value corresponds to one of the three variables represented in the final three-dimensional spectrum produced after each measurement [29,30]. Then, the analytes enter the IMS section of the device. Here, they are ionized by an ionization source. This source can either be a radioactive source, like Tritium or Nickel sources, or an X-ray source [31]. Once ionized, the formed ions are exposed to a weak and homogeneous electric field that is responsible for inducing velocity. The velocity of each ion is ordinarily called the drift velocity, vd. Once accelerated, the ions drift throughout the entire drift tube of the IMS, and, at the end of the tube, they are detected at specific temporal instants. The time required to cross the entire tube is, as for the GC, a compound-specific value commonly known as the drift time, dt [28,30]. Figure 2 schematizes the entire GC–IMS analysis, from the injection of the sample previously stored in the syringe-based containers, to the final detection using the Faraday plate detector.

As mentioned, both the retention and drift times are values characteristic of each analyte; in this way, they can be used for identification purposes. Nonetheless, it is common practice to calculate another compound-specific constant. This constant, defined as the ion mobility constant, *K*, translates the relationship between the drift time, *D_t_*, the magnitude of the electric field, *E*, and the length of the drift tube, *L* [28,32]. *K* can be calculated as follows:

(1) $$K=\frac{v_{d}}{E}=\frac{L}{E\cdot D_{t}},$$

Since the ion mobility constant depends on the pressure and temperature conditions existent during the analysis, it is common practice to normalize it to standard environmental values of pressure and temperature [28,32]. The normalized ion mobility constant, *K*_0_, can be calculated as follows:

(2) $$K_{0}=K\frac{P}{P_{0}}\frac{T_{0}}{T},$$

**Figure 3:**
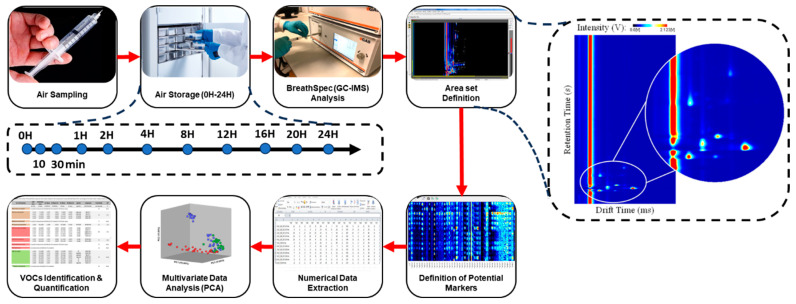
Representative scheme of the methodology performed during the study (**left**), and example of a GC–IMS spectrum (**right**).

Once the GC–IMS measurement is concluded, a three-dimensional spectrum is produced. Two of the three coordinates represent both the retention and drift times in seconds and milliseconds, respectively. The third coordinate corresponds to the intensity (V) of each compound detected during the analysis. The intensity can be directly related to the concentration that each analyte used to have in the original sample, and can be used for quantification purposes [15,33]. A generic spectrum produced after the analysis of environmental air is illustrated in Figure 3. Here, the drift and retention times are, respectively, represented in the x and y axes, and the intensity is represented using a color scale. An enlarged section is equally included for visualization purposes.

#### 2.3.2. Analysis Settings and Parameters

Air samples were analyzed using a BreathSpec^®^ apparatus manufactured by G.A.S., GmbH (Dortmund, Germany), which is an analytical system that combines Gas Chromatography (GC) and Ion Mobility Spectrometry (IMS). The IMS contains an ionization source of tritium (^3^H—β radiation, of 300 MBq) and a drift tube 98 mm in length. The chromatographic column, an MXT-200, of the GC–IMS device has the following dimensions: 30 × 0.53 × 1 (length (m) × internal diameter (mm) × thickness (μm)) and a mid-polar stationary phase of trifluoropropylmethyl polysiloxane. More detailed technical specifications of the used equipment are presented in Table 1.

For the analysis using the GC–IMS, a 500 V/cm electric field was applied inside the drift tube, positively polarized. The equipment was also connected to a Circular Gas Flow Unite (CGFU) from the same manufacturer, which supplies purified air as the carrier and drift gases. The gas flows were 150 mL/min for drift, and 10–150 mL/min for the carrier flow, respectively, for 300 s of each experimental run. More detailed conditions of the analysis are presented in Table 2.

### 2.4. Data Analysis and Data Validation

All GC–IMS three-dimensional spectra were processed using the LAV software (version 2.2.1.—G. A. S. Dortmund, Germany). In total, 82 analytes were detected for all observed peaks, thus extracting the values of the drift and retention times, and the normalized intensity variation. As a data pretreatment step, the exported intensity variation was normalized before the PCA by performing a background subtraction (i.e., removing intensity values from the room air measurements directly measured using the GC–IMS device without collection or storage in air containers). Afterwards, normalized intensity variations were statistically processed using partial least squares (PLS) regression and principal component analysis (PCA) to quantify the differences between the samples stored in each type of container at different storage times. IBM SPSS Statistics for Windows, version 23 (IBM Corp., Armonk, NY, USA) was used for such data analysis. 

The application of data validation approaches to chromatographic and spectrometric data plays a critical role in thoroughly assessing cause–effect systems [34,35,36] and thus allows both to assess the effects of syringe-based containers on VOC samples. This ultimately enhances the confidence and reliability of cause-–effect analyses in various scientific fields. In this scope, the repeatability of the measurements performed for each type of container and storage time was evaluated via the comparison of 3 replicates, where precision and standard deviations (for both D_t_ and R_t_) were evaluated (see results in Section 3.1). VOC identification was not performed for all GC–IMS intensity maxima, as the aim of the study was to compare the suitability of three distinct containers for the long-term storage and preservation of gas samples. Instead, only 4 VOCs were identified for sample reproducibility evaluation.

Figure 3 schematizes the set of procedures (from air sampling to data analysis) applied during this study and mentioned before. 

## 3. Results and Discussion

### 3.1. Sample Repeatability

A total of 99 spectra were collected from measurements conducted using eleven different storage times (according to Figure 3). The repeatability of the measurements performed for each type of container and storage time was assessed via a comparison of three replicates. The precision and analytical performance of the method were evaluated by statistically analyzing the repeatability of the GC–IMS data, including the normalized intensity for all analytes, and the retention time and relative drift time for four randomly selected VOCs (i.e., ethanol, isopropanol, acetone and propanal).

The VOC patterns detected from the analysis of the samples with the same storage for each container type were similar between the three replicates. The mean value and the standard deviation of the total intensity of the analytes detected in the samples immediately analyzed were 4.5 ± 0.1, 4.1 ± 0.3 and 4.1 ± 0.3 (V) for the plastic syringe with AC1, AC2 and AC3, respectively. This indicates a mean relative error of 5.61% for the normalized intensities of all analytes. Table 3 presents the mean retention and drift times (and corresponding standard deviation values, SD) for the four randomly selected analytes.

The overall relative error was found to be up to 0.46% for the retention time, 0.06% for the relative drift time, and 5.61% for the normalized intensity, which indicates the high precision of the data collected using the BreathSpec. These overall values were calculated using the mean relative error values for the four VOCs selected. Of note, the SD of the retention time for acetone was found to be 0.0 s due to no variations occurring in the acetone retention time for all nine replicates (i.e., three replicates of three syringe-based ACs). This careful adherence to the specified conditions confirms that the collection procedure is effective in preserving air samples. Without such adherence, the standard deviation for the average values of normalized intensity would be significantly higher, rendering the data statistically invalid and non-repeatable.

**Figure 4:**
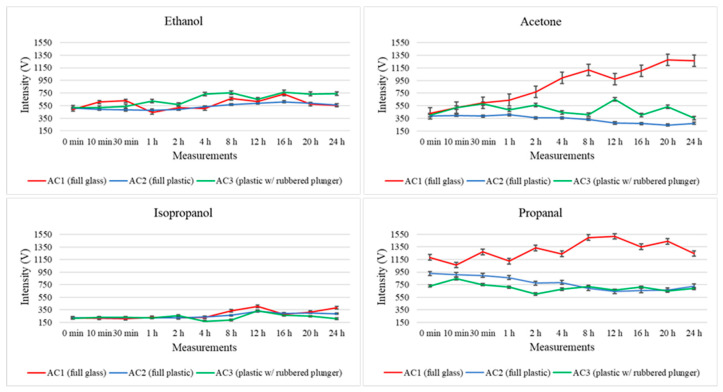
Variations in the total intensity levels registered for four randomly selected analytes (ethanol, acetone, isopropanol and propanal) throughout the 24 h study for the three types of storage containers (AC1: fully glass syringe, AC2: fully plastic syringe, AC3: plastic syringe with rubbered plunger).

Regarding the third value registered in the three-dimensional spectrum produced during the GC–IMS measurement, Figure 4 illustrates the total intensity registered for ethanol, acetone, isopropanol and propanal after each one of the considered storage times. Here, the intensity levels are represented in red, blue and green, respectively, for the full glass syringe (AC1), full plastic syringe (AC2), and plastic syringe with a rubbered plunger (AC3).

Further considerations regarding the intensity variation are discussed below; nonetheless, the proposed method demonstrates excellent performance for VOC analysis using GC–IMS. In fact, the low standard deviation observed between consecutive measurements further confirms the precision (and therefore repeatability) of the method and highlights the suitability of this technology for analyzing air samples. Therefore, the variability between replicates indicates that both sampling and analysis procedures applied in this study are reliable and consistent, denoting the stability of measurements per condition evaluated.

### 3.2. Sample Degradation and Compositional Variation

**Figure 5:**
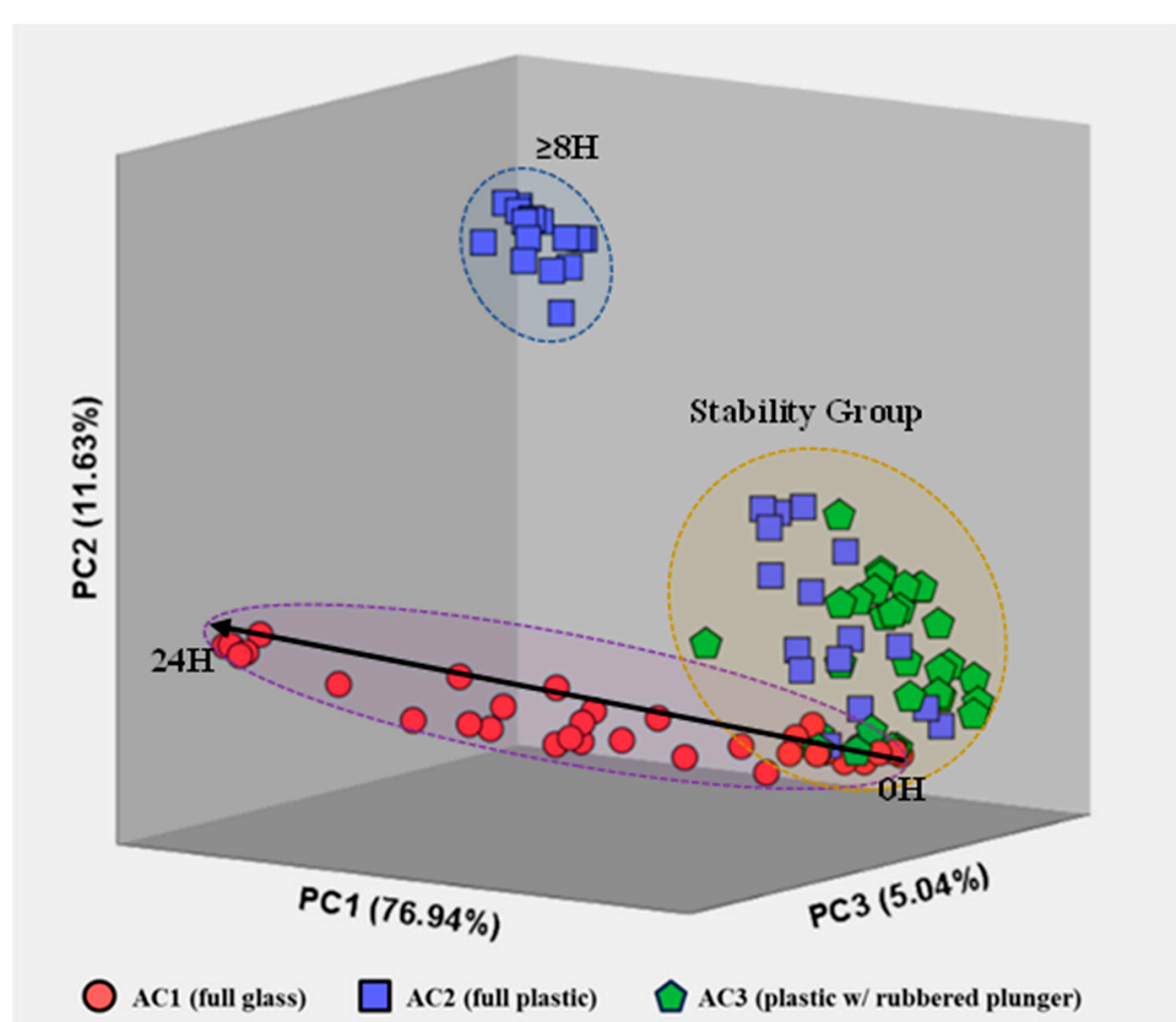
Representation of AC1 (circles), AC2 (squares) and AC3 (triangles) measurements for all storage times after PCA, including the three most relevant principal components. The total variance is also included for each principal component.

Although a comparison of the visual spectra provides a qualitative assessment of the most concentrated compounds, proper statistical data analysis allows us to perceive hidden (or not so visible) evidence and differentiate patterns in the air samples stored for different times using the three container types. The application of unsupervised PCA (Figure 5) highlights the differences between the profile of air stored in glass syringes (AC1, circles), fully plastic syringes (AC2, squares) and plastic syringes with a rubbered plunger (AC3, triangles). 

Principal Components (PC) exhibited 76.94% [PC1], 11.63% [PC2] and 5.041% [PC3] of the total explained variance in the data set for all measurements of each air sample, resulting in a total explained variance of 93.61%. Besides the high repeatability between replicas (i.e., samples with the same storing conditions: container type and storage time), Figure 5 also evidences a cluster containing measurements of the three container types for short storage times (t < 4 h), i.e., a stability group. Such results indicate that all three container types are suitable to use within this timeframe without significant changes in the VOC profile of environmental air samples, establishing a stability group in the PCA plot.

Additionally, the VOC emissions for the AC1 air samples follow a horizontal evolution along the PC1. Such behavior suggests a progressive change in the VOCs emitted from the air samples stored in these glass containers with an increasing storage time. This directly contrasts with the results obtained for AC2 (fully plastic syringes), where an abrupt change in VOC emissions occurs for storage times >8 h. Contrarily, measurements for air samples stored in plastic syringes with rubbered plungers were all constrained within the stability group.

The overall separation of VOC emissions, within the stability group, is caused mainly by usual compositional fluctuations in the air samples and by equipment uncertainty. Meanwhile, air measurements located outside the stability group may be caused by sample degradation after a certain storage time. Specifically, AC1 appears to have a higher and progressive degree of degradation over time, AC2 has an abrupt change in VOC emissions after 8 h of storage and AC3 indicates that the samples are preserved independently of the storage time (within the 24 h included in the study).

Moreover, the behavior of the samples stored in each type of container seems to have a common location of origin in the graph (within the stability region). To further understand the behavior of the air samples stored in the three container types over the storage time considered, new score plots were constructed for each type of container.

#### 3.2.1. Air Storage in Glass Syringe with Glass Plunger (AC1)

**Figure 6:**
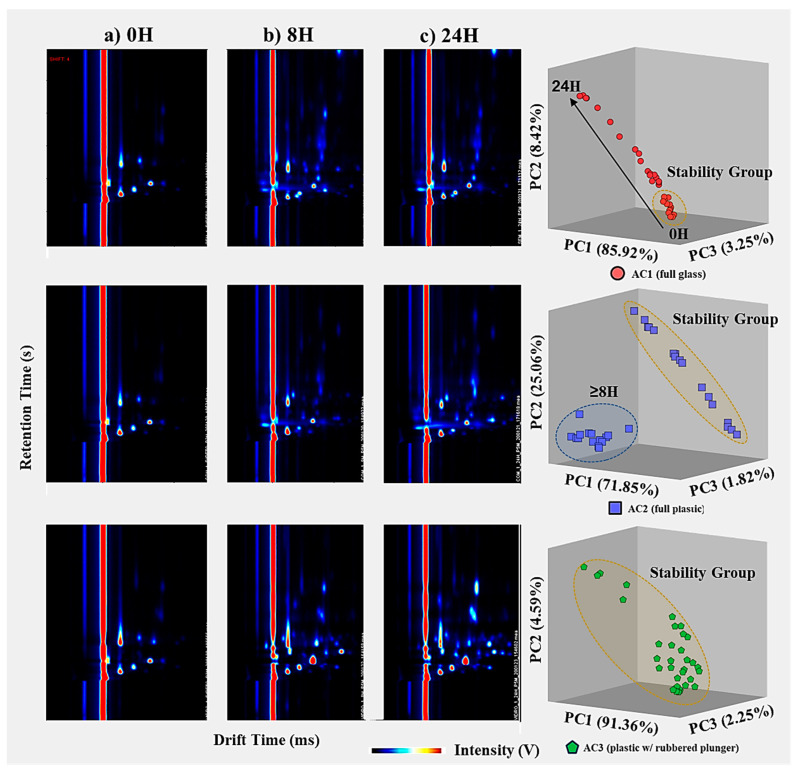
Temporal comparison of air measurements stored using AC1 (**top**), AC2 (**middle**) and AC3 (**bottom**) for a total storage time of 24 h; (**left**) three representative GC–IMS spectra for air samples analyzed (**a**) directly after the collection, (**b**) after 8 h of storage, and (**c**) after 24 h of storage; (**right**) PCA plot score demonstrating two distinct VOC profiles for air samples stored over 8 h, for the three replicates (1st blue, 2nd red and 3rd green).

Significant changes in the VOC emission profile are observed when comparing spectra from three storage times: 0 h (short-term), 8 h (intermediate), and 24 h (long-term). The samples analyzed without storage (0 h) exhibit the lowest number of VOCs compared to the other timeframes. In particular, at 8 h of storage, there are significant changes in the VOC emission profiles, characterized by the presence of new intensity peaks in the spectra, indicating the detection of new analytes. From 8 h to 24 h of storage, the VOC emission spectra show an increase in intensity for the newly detected peaks after 8 h, as well as the identification of a novel set of peaks/VOCs. This observation is further supported by PCA, which accounts for 97.59% of the total explained variance for the three most relevant principal components. The PCA analysis demonstrates a progressive linear distribution over time, as depicted in Figure 5 and confirmed in Figure 6. Figure 6 includes three exemplar spectra for air samples analyzed without storage (0 h), after 8 h, and after 24 h of storage, as well as a PCA score plot representing all measurements across all storage containers.

Since glass is an inert material, it would be expected to have an increased capacity to maintain stable VOC profile emissions throughout the 24 h in the air container. While the results initially support this expectation, especially for no storage (0 h), where the spectra are most similar to blank measurements, the same conclusion cannot be drawn for longer storage times (>1 h). This discrepancy may be attributed to two possible factors: (a) potential coating treatments applied to glass surfaces that reduce friction during the actuation of the plunger, and/or the (b) higher reactivity of VOCs in glass due to their weaker ability to prevent light effects. Both hypotheses suggest the generation of new peaks/VOCs via chemical interactions between the container material and the analytes in the air sample.

#### 3.2.2. Air Storage in Plastic Syringe with Plastic Plunger (AC2)

When comparing the spectra for the three different storage times (0 h, 8 h, and 24 h), significant changes are observed in the emission profile of VOCs. The spectra of samples analyzed without storage (0 h) exhibit the lowest number of VOCs compared to the other timeframes. At 8 h of storage, there are noticeable alterations in the VOC emission profile with the emergence of new intensity peaks in the spectra, indicating the detection of new analytes. However, no significant changes in the VOC emissions (i.e., same peaks detected) occur between 8 h and 24 h of storage time, only an increase in the intensity or concentration of the VOCs. The application of PCA solely for the air samples stored in AC2, with 98.73% of the total explained variance for the three most relevant principal components, clearly demonstrates the abrupt difference in the VOC emission profiles after 8 h, as depicted in Figure 5 and confirmed in Figure 6.

The appearance of new VOCs in the spectra of samples stored for more than 8 h might be explained by, over time, the initial compounds stored in AC2 being degraded, forming secondary VOCs. Contamination by external VOCs, exogenous or emitted from the plastic, can also explain the increase in VOCs detected, thus indicating the inability to store air samples for more than 8 h. This might also be linked to the poor isolation of the plastic plunger after this period.

#### 3.2.3. Air Storage in Plastic Syringe with Rubber Plunger (AC3)

In contrast to AC1 and AC2, the pattern of the analytes detected in the air samples stored in AC3 remains relatively consistent over the 24 h study period. The analytes detected in the samples without storage (blanks and 0 h) exhibit similar intensity levels to those detected in the air samples stored for different timeframes up to 24 h, with only minor intensity increases observed over consecutive 8 h periods. This phenomenon is mainly attributed to the inevitable slow degradation of these compounds.

Furthermore, this observation is supported by the PCA results, with 98.19% of the total explained variance accounted for by the three most relevant principal components (as shown in the bottom images of Figure 6), displaying a distinct cluster within the stability group (as depicted in Figure 5). This indicates that regardless of the storage time in the studied container, the samples maintain their main features without significant degradation or contamination by external analytes. In the case of the PCA for the AC3-stored air samples, the points are closely clustered together, except for five measurements (out of 33) that fall outside this group. However, these five measurements have irregular storage times of 30 min, 2 h, 12 h (twice), and 20 h. The randomness in the storage times for these samples suggests irregularities specific to these samples, unrelated to the storage capabilities of AC3.

Therefore, AC3 has proven to be significantly better at preserving the characteristics of the original sample and avoiding influences from external factors (both environmental and those related to AC3 materials) over extended storage periods, even when considering potential contaminations from the VOC emission profile of the rubbered plunger.

## 4. Conclusions

The storage of gaseous samples plays a crucial role in preserving the integrity and stability of volatile organic compounds (VOCs) for various applications, such as disease biomarker research, metabolite detection, and air quality control. This study evaluated the suitability of three commercially available syringes (AC1, AC2, and AC3) commonly used as air containers for sample collection, storage, isolation, and transportation. 

The application of GC–IMS as the analytical technique has proven to be highly suitable for evaluating air samples stored over both short and long periods, thus assessing the preservation capabilities of air containers (ACs). GC–IMS stands out for its exceptional selectivity, sensitivity, and analytical flexibility, combined with its portability and ability to perform longitudinal studies of volatile samples.

This study demonstrated the good repeatability of the measurements made and identified differences in the VOC profiles based on the storage time and container type. The analysis of the data using unsupervised PCA provided deeper insights into the overall VOC emission profile of air samples stored for different times using the three container types. Samples stored in glass syringes (AC1), fully plastic syringes (AC2), and plastic syringes with rubbered plungers (AC3) exhibited different behaviors over time.

Although all air containers can be used in short-term analysis (up to 1 h), glass syringes have the clearest measurements. Therefore, glass syringes proved to be suitable for direct analyses, but inappropriate for medium- and long-term storage scenarios. For long-term storage, AC3 proved to be the most appropriate for sample preservation. 

Despite lacking the confirmation of these findings for more complex volatile samples (e.g., breath samples or even room air samples from environments with high humidity levels), this study provides crucial insights into the selection of appropriate syringe-based containers and their impact on VOC stability. These are imperative considerations for the selection of suitable containers for the transport and storage of VOC samples and, consequently, a reliable and insightful analysis of air-based samples. 

## Figures and Tables

**Table 1:** GC–IMS equipment (BreathSpec^®^) specifications.

Parameters	Values	Units
Sample Loop Volume	1	mL
GC Column Model	MXT-200	–
GC Column Length	30	m
GC Column Diameter	0.53	mm
Ionization Source	Tritium—β Radiation	–
Ionization Intensity	300	MBq
Drift Region Length	9.80	cm
Drift Potential Difference	5	kV
IMS Pressure Range	757–760	Torr
Electric Field Intensity	500	V/cm
Resolving Power Range	65–70	–

**Table 2:** Analysis conditions applied for this assessment.

Parameters	Values	Units
Carrier Gas	Purified Air	–
GC Temperature	343.15	K
Ionization Polarity	Positive	–
IMS Temperature Range	297.15–301.15	K
Carrier Gas Flow	10–150	mL/min
Drift Gas Flow	150	mL/min
Analysis Duration	300	s

**Table 3:** Mean retention and drift times (and corresponding standard deviation values) for the four randomly selected analytes.

Compound	Rt (s)	SD Rt (s)	D_t_ (RIP Rel.)	SD D_t_ (RIP Rel.)
Ethanol	24.2	0.1	1.0617	0.0005
Isopropanol	25.6	0.1	1.1083	0.0006
Acetone	27.5	0.0	1.1650	0.0008
Propanal	33.2	0.2	1.0626	0.0006

## Data Availability

The data presented in this study are available on request from the corresponding author. The data are not publicly available due to confidentiality rights established between funding institutions.
